# High-Entropy Borides under Extreme Environment of Pressures and Temperatures

**DOI:** 10.3390/ma15093239

**Published:** 2022-04-30

**Authors:** Seth Iwan, Chia-Min Lin, Christopher Perreault, Kallol Chakrabarty, Cheng-Chien Chen, Yogesh Vohra, Rostislav Hrubiak, Guoyin Shen, Nenad Velisavljevic

**Affiliations:** 1 Department of Physics, University of Alabama at Birmingham, Birmingham, AL 35294, USA; iwanseth@uab.edu (S.I.); lincm@uab.edu (C.-M.L.); cperre@uab.edu (C.P.); kallol89@uab.edu (K.C.); chencc@uab.edu (C.-C.C.); 2 High Pressure Collaborative Access Team (HPCAT), X-ray Science Division, Argonne National Laboratory, Argonne, IL 60439, USA; hrubiak@anl.gov (R.H.); gyshen@anl.gov (G.S.); velisavljevi1@llnl.gov (N.V.); 3 Physics Division, Lawrence Livermore National Laboratory, Livermore, CA 94550, USA

**Keywords:** high pressure, high temperature, high-entropy materials, computational simulations, compressive strength

## Abstract

The high-entropy transition metal borides containing a random distribution of five or more constituent metallic elements offer novel opportunities in designing materials that show crystalline phase stability, high strength, and thermal oxidation resistance under extreme conditions. We present a comprehensive theoretical and experimental investigation of prototypical high-entropy boride (HEB) materials such as (Hf, Mo, Nb, Ta, Ti)B_2_ and (Hf, Mo, Nb, Ta, Zr)B_2_ under extreme environments of pressures and temperatures. The theoretical tools include modeling elastic properties by special quasi-random structures that predict a bulk modulus of 288 GPa and a shear modulus of 215 GPa at ambient conditions. HEB samples were synthesized under high pressures and high temperatures and studied to 9.5 GPa and 2273 K in a large-volume pressure cell. The thermal equation of state measurement yielded a bulk modulus of 276 GPa, in excellent agreement with theory. The measured compressive yield strength by radial X-ray diffraction technique in a diamond anvil cell was 28 GPa at a pressure of 65 GPa, which is a significant fraction of the shear modulus at high pressures. The high compressive strength and phase stability of this material under high pressures and high temperatures make it an ideal candidate for application as a structural material in nuclear and aerospace fields.

## 1. Introduction

In a search for novel materials that retain their mechanical properties and oxidation resistance at high temperatures, transition metal borides have traditionally attracted increased attention [1]. This field of ultrahard and refractory materials has seen a new impetus recently due to the advent of high entropy materials that contain a random solid solution of five or more constituent elements [2,3,4]. Thus, one can envision a variety of high-entropy alloy, oxide, carbide, nitride, and boride materials that are stabilized by the high entropy of mixing given by ΔSmix = R ln(N), where R is the gas constant and N is the number of constituent elements. This high entropy of mixing also ensures that these materials are stable at high temperatures due to lower Gibb’s free energy and can be exploited in applications where phase stability is an important materials selection criterion. Several high-entropy transition metal borides were synthesized recently [5], and their hardness and oxidation resistance were shown to be higher/better than the average performances of five individual metal diborides made by identical fabrication processing. Our motivation in this paper is to examine the structural and mechanical properties of high-entropy borides (HEBs) under combined high-pressure and high-temperature conditions, where materials synthesis and structural/compressibility measurements are combined in a single experiment [6]. In our earlier study on the synthesis of (Hf, Mo, Zr, Ta, Ti)B_2_ material (reported in [6])_,_ the high-pressure high-temperature synthesized sample was not recovered at ambient conditions and, hence, only limited information on morphological, structural, element mapping, and mechanical properties could be gathered. We have addressed these shortcomings in the present study, where synthesized HEB samples have been recovered at ambient conditions for precise lattice parameter measurements, microstructure, characterization of residual phases, and subsequent shear strength measurements at high pressures in a diamond anvil cell. In addition, while a great deal of experimental data has been collected on HEB materials, the fundamental understanding of their entropy forming ability and structural and mechanical properties is still lacking due to difficulty in modeling disordered materials. We present a comprehensive theoretical and experimental investigation of prototypical high-entropy boride (HEB) materials such as (Hf, Mo, Nb, Ta, Ti)B_2_ and (Hf, Mo, Nb, Ta, Zr)B_2_ under combined high pressures and high temperatures and offer a detailed structural and mechanical characterization of these materials recovered from extreme environments. The (Hf, Mo, Nb, Ta, Ti)B_2_ material was chosen for detailed high-pressure, high-temperature investigation because it is known to have the best thermal oxidation resistance of all the known transition metal borides up to 1200 °C [5], and our preliminary mechanical property measurements indicate that (Hf, Mo, Nb, Ta, Zr)B_2_ has a high hardness value amongst HEB materials. In this study, we present a novel experimental approach where HEB material is synthesized under high pressures and high temperatures and its crystal structure and compressibility are studied under extreme environments in the same sample chamber without exposing the sample to ambient conditions, thereby minimizing contamination and generating data on high-purity HEB materials.

## 2. Materials and Methods

We performed density functional theory (DFT) calculations using the VASP software [7,8], which is based on a pseudopotential and plane-wave basis. We adopt the projector augmented wave (PAW) method [9,10] and the generalized gradient approximation (GGA) function based on Perdew-Burke-Ernzerhof (PBE) formalism [11]. The cutoff energy for the plane-wave basis was 446 eV, and the k-points were sampled by a Γ-centered Monkhorst-Pack mesh with a k-point density per reciprocal atom (KPPRA) ~10,000. The convergence criteria of electronic self-consistency and structural relaxation were set to 10−6 eV/unit cell and 10−3 eV/Å, respectively.

To model high-entropy materials, we further employed the stochastic (or special) quasi-random structure (SQS) [12]. SQS structures are traditionally generated by exhaustively enumerating all possible occupations of the sites in a supercell. However, its computation complexity grows exponentially with the supercell size, thereby suitable only for small unit cells (a few tens of atoms). Here, we utilize stochastic Monte Carlo approaches implemented in the Alloy Theoretic Automated Toolkit (ATAT) “mcsqs” code [13], which allows efficient generation of SQS structures over 100 atoms. After the SQS construction, we fully relaxed the structures under pressure up to 70 GPa. Afterwards, we further utilized the strain–stress method [14] implemented in VASP to compute the elastic constants Cij, from which the bulk modulus K and shear modulus G can be evaluated using the Voigt-Reuss-Hill (VRH) average [15]. We note that the crystal symmetry of the original parent lattice can be reduced in SQS due to the presence of different atoms. In this case, a symmetrized Cij, averaged over different directions, can be applied [16].

The high-pressure, high-temperature synthesis of HEB (Hf, Mo, Nb, Ta, Ti)B_2_ and (Hf, Mo, Nb, Ta, Zr)B_2_ samples was carried out at HPCAT beamline 16-BM-B [17] using a large volume Paris-Edinburgh (PE) press with a type of Cupped Drickamer Toroidal (CDT) cell. The synthesis process employed metal oxide precursors mixed with boron carbide and graphite that were high-energy ball-milled (Spex 8000M). CDT assembly is the newer cell being implemented with PE press at High Pressure Collaborative Access Team (HPCAT), and it provides more uniform (hydrostatic) pressure conditions and allows for extending experiments to higher pressure conditions (up to ~10 GPa). The beamline provides an in situ energy-dispersive X-ray diffraction (EDXD) probe during the synthesis process as well as subsequent pressure–volume measurements to 9.5 GPa and 2273 K. The pressures were calibrated at high temperatures using the thermal equation of state for magnesium oxide [18] in the sample assembly, while the temperatures were established in a separate calibration of thermocouple measurement experiments and the power delivered to the graphite heater. The shear strength measurements on the (Hf, Mo, Nb, Ta, Ti)B_2_ sample were carried out at HPCAT beamline 16-BM-D using radial angle-dispersive X-ray diffraction in a panoramic diamond anvil cell [19]. The secondary electron SEM images were acquired with a Quanta FEG 650 scanning electron microscope.

## 3. Results

### 3.1. Theoretical Results

Studying the mechanical properties of HEAs and HEBs is computationally challenging due to the extremely large phase space of possible compositions and systems that can be formed. It would be convenient if the elastic properties could be obtained using calculations on one single large-size supercell that effectively captures short-range correlation and efficiently mimics the randomness of high-entropy effects. Such approaches do exist, and we employ the SQS [12] approach in this paper. SQS represents the best periodic supercell that approximates a true disordered state for a given number of atoms. This is achieved by matching a specified set of correlations between neighboring sites to the corresponding correlation of a fully disordered state in the thermodynamic limit.

**Figure 1:**
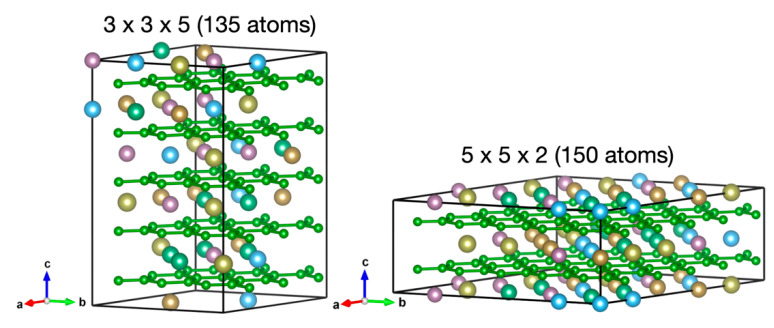
Special quasi-random structures (SQSs) for simulating the compression and mechanical behaviors of the hexagonal high-entropy boride (Hf, Mo, Nb, Ta, Ti)B_2_. The SQS structures include 135 atoms (**left**) and 150 atoms (**right**), which, respectively, can be regarded as 3 × 3 × 5 and 5 × 5 × 2 supercells of the parent lattice of the hexagonal AlB_2_ structure.

Figure 1 shows the SQS structures generated by Monte Carlo methods implemented in the ATAT-mcsqs code [13] for the hexagonal HEB (Hf, Mo, Nb, Ta, Ti)B_2_. These structures of 135 and 150 atoms can be respectively regarded as 3 *×* 3 × 5 and 5 × 5 × 2 supercells of the parent lattice of hexagonal AlB_2_ structure, but with the Al Wyckoff site replaced by five different metals with equal probability. ATAT-mcsqs allows the efficient generation of SQS structures over 100 atoms, and it is important to check different SQS sizes and shapes to ensure that physical properties of interests are converged and represent those in the thermodynamic limit. 

**Figure 2:**
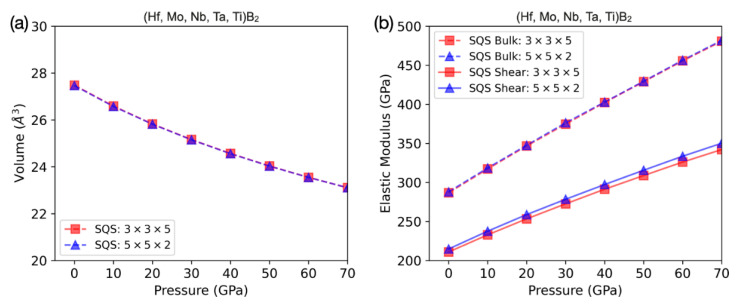
Volume and elastic moduli of different SQS structures computed for (Hf, Mo, Nb, Ta, Ti)B_2_. (**a**) The computed volumes of the 135-atom (red square) and 150-atom (blue triangle) SQS structures match well with each other. (**b**) The bulk and shear moduli computed by both SQS structures are also in very good agreement. The theoretical bulk and shear moduli at zero pressure from the 150-atom SQS structure are K_0_ = 288 GPa and G_0_ = 215 GPa, respectively.

Figure 2a indicates that the volumes versus external pressure for different fully relaxed SQS structures agree extremely well. At ambient pressure, the volume of (Hf, Mo, Nb, Ta, Ti)B_2_ from the 150-atom SQS structure (blue curve) is 27.47 Å^3^. This theoretically predicted volume is within 2% of the experimentally measured value of 26.882 Å^3^. Figure 2b shows the corresponding bulk and shear moduli as a function of pressure. In the 150-atom SQS results (blue curves), the ambient-pressure bulk and shear modulus values are, respectively, K_0_ = 288 GPa and G_0_ = 215 GPa. The theoretical value of K_0_ is in good agreement with the experimentally determined K_0_ = 276 GPa.

### 3.2. Experimental Results

**Figure 3:**
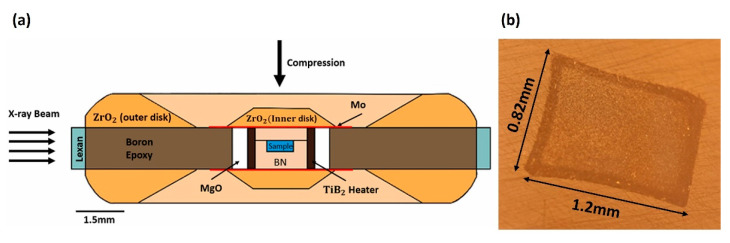
(**a**) Cupped Drickamer toroidal (CDT) employed in the high-pressure, high-temperature synthesis of high-entropy borides. The sample chamber contains a mixture of precursor materials depending on the desired composition. (**b**) The synthesized (Hf, Mo, Nb, Ta, Ti)B_2_ sample of 1.2 × 0.82 mm was recovered for a detailed characterization of its physical and mechanical properties, as described in the text.

The precursor materials contained equimolar amounts of five transition metal oxides: HfO_2_, TiO_2_, NbO_2_, Ta_2_O_5_, MoO_3_, Nb_2_O_5_, carbon black, and boron carbide from Alfa Aesar. First, the metal oxides combined with carbon were high-energy ball-milled (Spex 8000M) for two hours in a tungsten carbide container and media. At hourly intervals, the ball mill was allowed to cool for 10 min. The boron carbide was then added to the mixture to blend by ball mill with the milled metal oxides and carbon. To reduce contamination of tungsten carbide, the mixture with B_4_C was wet-milled in acetone for four hours with zirconia balls. The mixture included an excess of B_4_C (9% by weight) to account for the boron lost due to the formation of boron oxide and carbon monoxide, B_2_O_3_ and CO, during high-pressure, high-temperature synthesis [20]. The milled mixture was dried before being passed through a 200-mesh sieve to improve the uniformity of particle size for subsequent processing. Figure 3 shows the sample assembly and the synthesized sample recovered after the synthesis at high pressures and high temperatures. 

**Figure 4:**
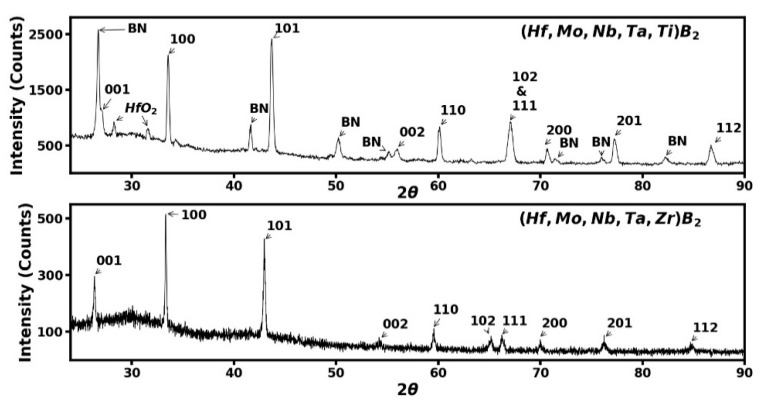
The angle-dispersive X-ray diffraction spectra for the two samples at ambient conditions after synthesis at high pressures and high temperatures. Both samples show a single hexagonal AlB_2_ phase with ten diffraction peaks labeled with (*hkl*) values; no contaminants were detected. The peaks marked BN come from the sample container used in the synthesis process and very weak unreacted hafnium oxide in the upper panel.

The sample recovered at ambient conditions after high-pressure, high-temperature synthesis was analyzed by angle dispersive X-ray diffraction (ADXD) to confirm that the single hexagonal AlB_2_ phase was observed with no WC-Co or ZrO_2_ contamination. Figure 4 shows ADXD patterns for both (Hf, Mo, Nb, Ta, Ti)B_2_ and (Hf, Mo, Nb, Ta, Zr)B_2_ samples, showing the single hexagonal AlB_2_ phase with no contaminants from the ball milling process. There is only a remanent BN phase from the sample container at the edges of the sample, along with a very weak amount of unreacted hafnium dioxide in one case. The measured ambient lattice parameters for (Hf, Mo, Nb, Ta, Ti)B_2_ are a = 3.0746 Å, c = 3.2837 Å, with unit cell volume V_0_ = 26.882 Å^3^. The measured ambient lattice parameters for (Hf, Mo, Nb, Ta, Zr)B_2_ are a = 3.1037 Å, c = 3.3822 Å, with unit cell volume V_0_ = 28.216 Å^3^.

**Figure 5:**
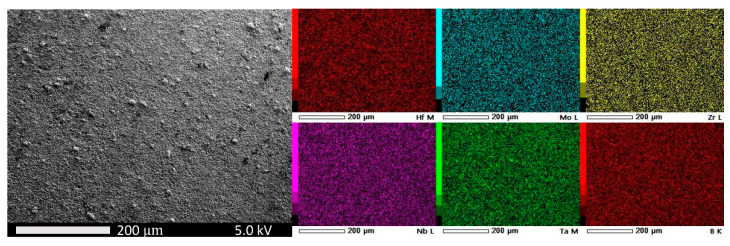
SEM and EDS analysis of high-pressure, high-temperature synthesized (Hf, Mo, Nb, Ta, Zr)B_2_ sample. The EDS analysis based on X-ray fluorescence shows the distribution of the five transition metals and boron in the high entropy boride sample.

**Figure 6:**
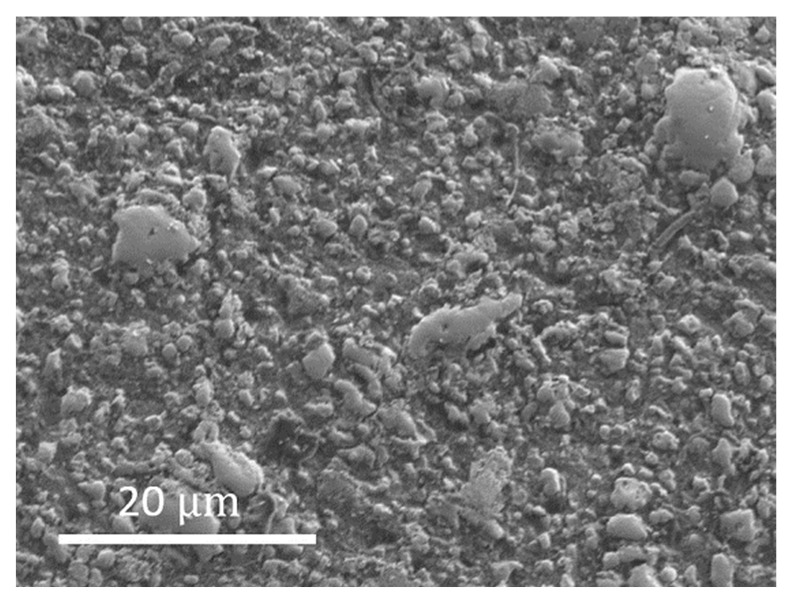
High-resolution SEM image of the (Hf, Mo, Nb, Ta, Zr)B_2_ sample showing an average grain size of 2 microns. The few large grains observed in this micrograph are also from the (Hf, Mo, Nb, Ta, Zr)B_2_ sample.

**Figure 7:**
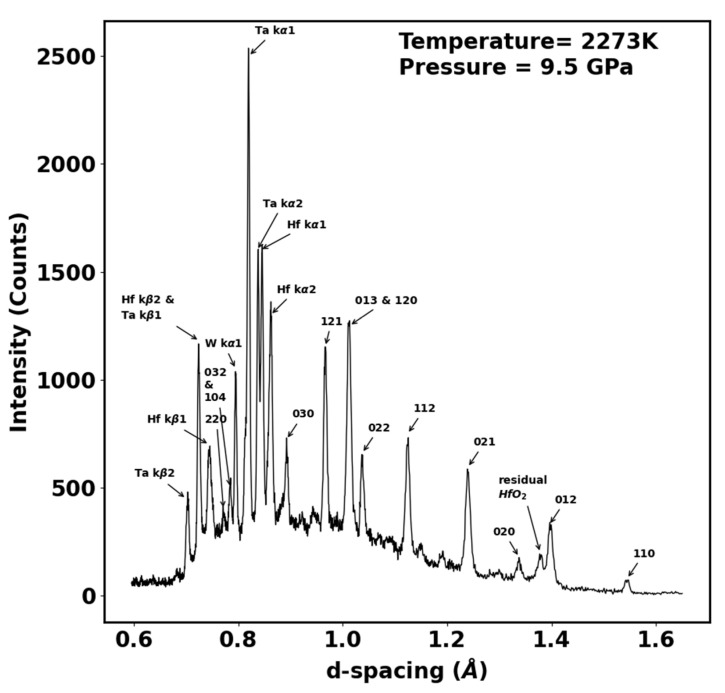
Energy-dispersive X-ray diffraction spectrum of the (Hf, Mo, Nb, Ta, Ti)B_2_ sample at a pressure of 9.5 GPa and temperature of 2273 K. The sample peaks are indexed to a hexagonal AlB_2_ phase, with lattice parameters described in the text, along with the fluorescence peaks from the constituent elements. A weak peak from residual precursor material hafnium dioxide is also indicated.

Scanning electron microscopy (SEM) and energy dispersive spectroscopy (EDS) were performed to ascertain the uniform distribution of elements in the high entropy boride. Figure 5 shows the SEM image and the EDS analysis showing the distribution of the five transition metals and boron throughout the bulk of the sample, confirming single-phase observation by X-ray diffraction. Further high-resolution EDS and nanoscale composition mapping studies will be needed to establish uniform compositions from nanoscale to microscale. The additional information on grain size is provided by a higher-resolution SEM image in Figure 6, which shows an average grain size of two microns. The high-pressure, high-temperature synthesized sample was studied up to 9.5 GPa and 2273 K, and the energy dispersive X-ray diffraction pattern is shown in Figure 7. The hexagonal ${\mathrm{AlB}}_{2}$ structure was stable to the highest pressure and temperature reached in this study, as evidenced by the strong (110), (012), (020), (021), (112), (022), (013)/(120), (121), and (030) distinct Bragg diffraction peaks from this structure, which are clearly visible in addition to the X-ray fluorescence peaks from the constituent elements (Figure 7). The measured lattice parameters for the hexagonal ${\mathrm{AlB}}_{2}$ phase at 9.5 GPa and 2273 K are a = 3.0886 Å and c = 3.2807 Å.

**Figure 8:**
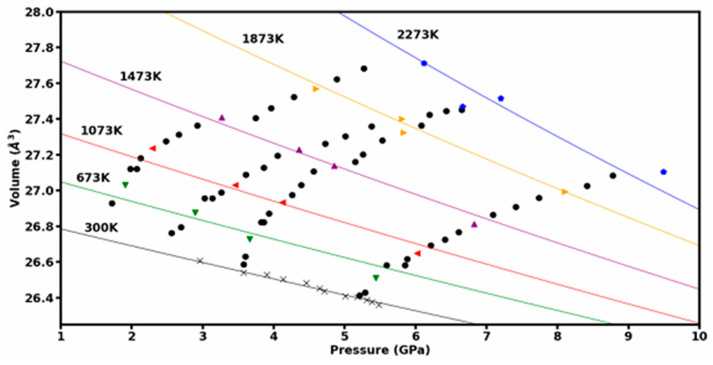
The measured Pressure–Volume–Temperature data for the (Hf, Mo, Nb, Ta, Ti)B_2_ sample to 9.5 GPa and 2273 K. The “x” marked data points were taken during decompression at 300 K. The solid lines are fitted to various isotherms ranging from 300 to 2273 K. The thermal equation of state fit is described in the text.

A series of high-temperature heating runs were conducted to map out the P-V-T space for this material. The decompression data was also collected at ambient temperature to establish the room-temperature equation of state. The combined data set is shown in Figure 8. The 2nd Order Birch Murnaghan equation of state (BM EoS) in Equation (1) was used with the thermal expansion model [21,22] shown in Equation (2) to extract the bulk modulus (*K*_0_), *dK*/*dT*, and the volumetric thermal expansion coefficient α.

(1) $$P\left(V\right)=\frac{3}{2}K_{0}\left[x^{\frac{7}{3}}-x^{\frac{5}{3}}\right]\left[1+\frac{3}{4}\left(K_{0}^{\prime }-4\right)\left(x^{\frac{2}{3}}-1\right)\right]$$

(2) $$V_{0T}=V_{00}\exp\left(\alpha_{0}\left(T-T_{ref}\right)+\frac{1}{2}\alpha_{1}\left(T^{2}-T_{ref}^{2}\right)+\alpha_{2}\left(\frac{1}{T}-\frac{1}{T_{ref}}\right)\right)$$

In Equation (1), *x = V*_0_*/V*, *K*_0_ is the bulk modulus, and *K*_0_*’* is the first pressure derivative. For Equation (2), the temperature-dependent volumetric thermal expansion α is described by coefficients α_0_, α_1_, α_2_ as *α = α*_0_* + α*_1_*T + α*_2_*T^−2^*. The fit to P-V-T data shown in Figure 8 yields information on the thermoelastic properties of the synthesized HEB sample. Using the ambient pressure unit-cell volume measured earlier V_0_ = 26.882 Å^3^, the fit resulted in a bulk modulus *K*_0_ = 276 GPa at ambient temperature with a fixed value of *K_0_’*= 4. The fitted value of the temperature derivative of bulk modulus is *dK/dT* = −0.090 GPa/K. Thermal expansion coefficients were calculated to be α_0_ = −1.533 × 10^−5^ *K^−1^*, α_1_ = 4.071 × 10^−8^ *K*^−2^, and α_2_ = 4.60 *K,* with the volumetric thermal expansion expressed as α = α_0_ + α_1_ *T* + α_2_ T^−2^ in the temperature range between 300 and 2300 K.

The radial X-ray diffraction data on the HEB sample was collected to 65 GPa using platinum as an X-ray pressure standard. Collected radial X-ray diffraction patterns were integrated into 72 azimuthal segments of δ = 5 degrees around the entire pattern using the methodology described in [19]. The measured d-spacing (dm) of the HEB sample for each 5-degree segment can be obtained using Equation (3):

(3) $$d_{m}\left(hkl\right)=d_{p}+d_{p}Q_{hkl}(1-3\cos^{2}\chi )$$

where *dp* is the hydrostatic component of compression, *Q_hkl_* is the lattice strain, and χ is the angle between the DAC compression axis and the diffraction plane normal defined by cosχ = cosδ cosθ. The linear relationship between measured d-spacing *d_m_* and the 1 *−* 3 cos^2^*χ* term allows for direct calculation of the estimated hydrostatic d-spacing *dp* by eliminating the directionally dependent lattice strain *Q_hkl_* term when 1 *−* 3 cos^2^*χ =* 0. When lattice strain *Q_hkl_* is present in a sample, the differential stress *t*, which is a measure of compressive yield strength [23] of the material, can be determined by averaging the strain over all *hkl* and using Equation (4). Furthermore, differential stress *t* is approximately twice the shear strength *τ* of the material.

(4) $$6\langle Q_{hkl}\rangle =\frac{t}{G},\;t=2\tau$$

where *G* is the sample shear modulus.

**Figure 9:**
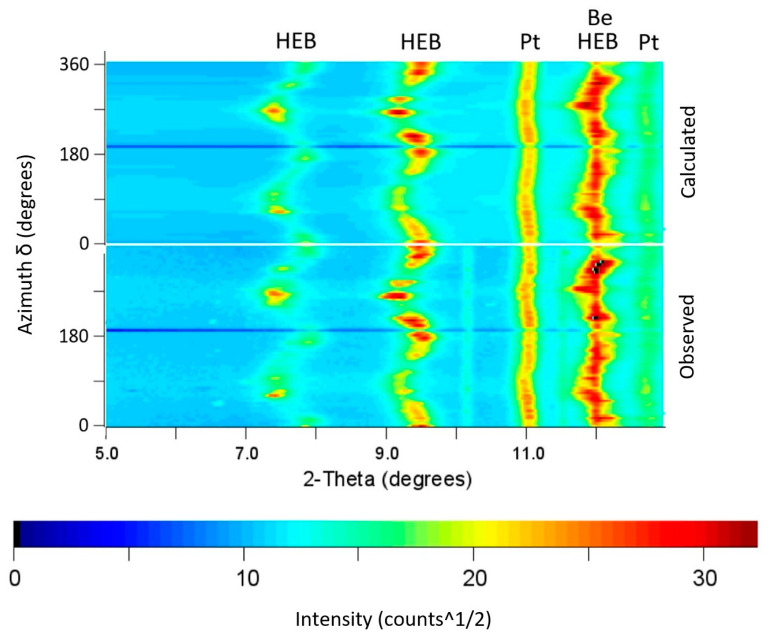
The X-ray diffraction pattern of the HEB sample (Hf, Mo, Nb, Ta, Ti)B_2_ along with platinum (Pt) marker at a pressure of 65 GPa in a radial diffraction mode with beryllium (Be) gasket. The waviness in the diffraction lines reflects the large anisotropy in elastic strains present for a high-strength material such as HEB compared to a platinum pressure marker. The lower panel shows the observed diffraction intensities in the azimuthal direction, while the upper panel shows the calculated intensities.

**Figure 10:**
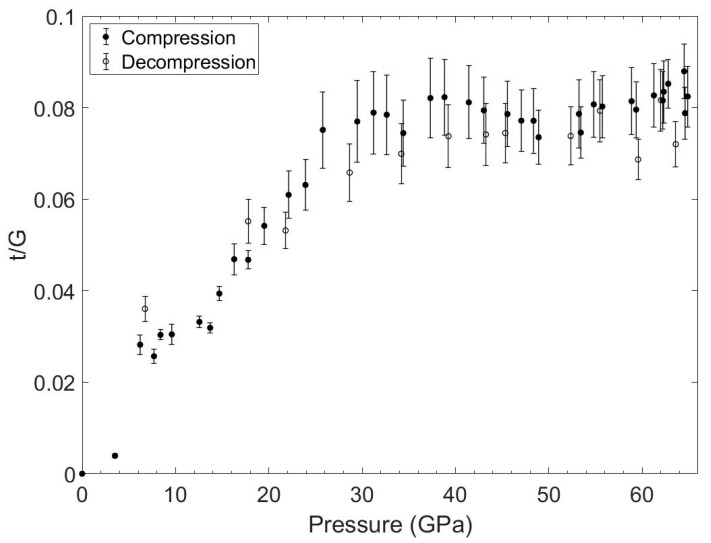
The measured value of differential stress (t) normalized to shear modulus for HEB (Hf, Mo, Nb, Ta, Ti)B_2_ sample to 65 GPa. The data are shown for both compression and decompression cycles. The HEB sample shows high differential stress, approaching 8% of the shear modulus.

Figure 9 shows the angle dispersive X-ray diffraction spectrum of the (Hf, Mo, Nb, Ta, Ti)B_2_ sample mixed with the platinum pressure marker at a pressure of 65 GPa. The variation in the 2-Theta diffraction angle along the azimuthal angle shows the effect of the differential stress supported by the sample. In contrast to the (Hf, Mo, Nb, Ta, Ti)B_2_ sample, the platinum pressure marker shows relatively less variation in the 2-Theta angle along the azimuthal angle. The lattice strain *Q_hkl_* is then averaged from the observed *hkl* peaks and used in Equation (4) to determine the compressive strength using the computed value of shear modulus. The differential stress *t* normalized to shear modulus (*G*) is plotted in Figure 10, with the hydrostatic pressure values obtained from the platinum marker. The experimentally measured differential stress *t* (or compressive strength) is observed to increase with increasing pressure and approaches a value as high as 8% of shear modulus at 65 GPa. Using the theoretical value of shear modulus at 65 GPa, the compressive strength of the (Hf, Mo, Nb, Ta, Ti)B_2_ sample is estimated to be as high as 28 GPa.

## 4. Discussion

The HEB samples were synthesized under high pressures and high temperatures, starting from ball-milled oxide precursors mixed with boron carbide and graphite powders. The reduction of grain size of precursor materials using ball-milling led to rapid transformation in several minutes to HEB under high pressures and high temperatures; however, transformation kinetics was not the focus of this study. The experimental approach, where HEB material is synthesized under high pressures and high temperatures and its crystal structure and compressibility are studied under extreme environments in the same sample chamber without exposing the sample to ambient conditions, has yielded crystal structure data, the thermal equation of state data, and compressive yield strength data on high-entropy transition metal boride materials. The hexagonal AlB_2_ phase of the high-entropy transition metal boride sample is stable to the extreme high pressures and high temperatures achieved in this study. The measured value of bulk-modulus for (Hf, Mo, Nb, Ta, Ti)B_2_ is *K_0_* = 276 GPa and is in good agreement with the theoretically predicted value of *K_0_* = 288 GPa in this study. The measured value of *K_0_* = 276 GPa for (Hf, Mo, Nb, Ta, Ti)B_2_ is lower than the reported value of *K_0_* = 344 GPa for (Hf, Mo, Zr, Ta, Ti)B_2_ [6]; however, the ambient pressure volume was not directly measured in the earlier study and that could impact the determination of bulk-modulus in [6]. The measured high compressive yield strength of HEB materials at high pressures is also supported by a theoretical model that predicts an increase in shear modulus with increasing pressure. The measured compressive yield strength of HEB is 8% of shear modulus (*G*) at 65 GPa. The shear strength τ is approximately half of compressive yield strength and is estimated to be about 0.04 *G* at 65 GPa. The theoretical estimate of τ is *G*/6 [19] or 0.167 *G* is four times higher than the experimental value. This difference is likely since crystal imperfections are not included in the theoretical models of HEB, and more realistic models are needed for better comparison with experimental shear strength data.

## 5. Conclusions

We present a comprehensive theoretical and experimental study of the compression and deformation behavior of prototypical high-entropy transition metal boride (Hf, Mo, Nb, Ta, Ti)B_2_ and (Hf, Mo, Nb, Ta, Zr)B_2_ samples to high pressures and high temperatures. The high-pressure, high-temperature synthesis and compression studies of high-entropy transition metal borides are carried out in the same sample chamber, thereby minimizing exposure to ambient environments. The hexagonal AlB_2_ phase of both high-entropy boride samples was found to be stable to pressure and temperature conditions of 9.5 GPa and 2273 K, respectively. The supercell approach using the stochastic (or special) quasi-random structure (SQS) predicts a bulk modulus value of 288 GPa, in excellent agreement with the measured value of 276 GPa. The changes in bulk modulus and thermal expansion coefficient with temperatures were also documented in this study. The compressive strength of the (Hf, Mo, Nb, Ta, Ti)B2 sample approaches a value of 28 GPa at an applied pressure of 65 GPa in a measurement carried out in a diamond anvil cell. The high compressive strength and phase stability of this material under high pressures and high temperatures make it an ideal candidate for application as a structural material in nuclear and aerospace fields.

## Data Availability

All data generated or analyzed and materials synthesized during this study are included in this published article. Additional raw data used and/or analyzed during the current study are available from the corresponding author upon reasonable request.
